# Relationship between bone density and bone metabolism in adolescent idiopathic scoliosis

**DOI:** 10.1186/s13013-015-0033-z

**Published:** 2015-03-19

**Authors:** Ko Ishida, Yoichi Aota, Naoto Mitsugi, Motonori Kono, Takayuki Higashi, Takuya Kawai, Katsutaka Yamada, Takanori Niimura, Kanichiro Kaneko, Hironori Tanabe, Yohei Ito, Tomoyuki Katsuhata, Tomoyuki Saito

**Affiliations:** Department of Orthopaedic Surgery, Yokohama City University Medical Center, 4-57 Urafune Minami-ku, Yokohama, Kanagawa 232-0024 Japan; Department of Orthopaedic Surgery, Yokohama City Brain and Stroke Center, 1-2-1 Takigashira Isogo-ku, Yokohama, Kanagawa 235-0012 Japan; Department of Orthopaedic Surgery, Yokohama City University, 3-9 Fukuura Kanazawa-ku, Yokohama, Kanagawa 236-0004 Japan

## Abstract

Several authors have confirmed that 27 to 38% of AIS patients had osteopenia. But few studies have assessed bone metabolism in AIS. This study assessed bone mineral density and bone metabolism in AIS patients using the bone metabolism markers, BAP and TRAP5b. The subjects were 49 consecutive adolescent AIS patients seen at our institutes between March 2012 and September 2013. Sixty-five percent of AIS patients had osteopenia or osteoporosis and 59% of AIS patients had high values for TRAP5b. The AIS patients with high values of TRAP5b had lower Z scores than those with normal values of TRAP5b. Higher rates of bone resorption are associated with low bone density in AIS patients.

The features of adolescent idiopathic scoliosis (AIS) have been researched. Since first reported by Burner et al. in 1982 [1], several authors have confirmed that 27 to 38% of AIS patients had osteopenia [2-6]. Furthermore osteopenia or osteoporosis had been reported to be one of causes of scoliosis curvature aggravation [6]. But few studies have assessed bone metabolism in AIS. Cheung et al. reported that the serum concentration of bone alkaline phosphatase (BAP, bone formation marker) in AIS patients was higher than that of controls, while urinary concentrations of deoxypyridinoline (bone resorption marker) in patients with AIS were lower [7]. Urinary deoxypyridinoline has been found to be more affected by renal function, fasting, and hourly variations than tartrate-resistant acid phosphatase serum band 5 (TRAP5b) [8].

The aim of this study was to clarify the bone metabolism in AIS and the relationship between bone metabolism and bone density in AIS. This study characterizes bone metabolism in AIS patients by assessing serum levels of TRAP5b.

## Subjects and methods

The subjects were 49 consecutive adolescent AIS patients seen at our institutes between March 2012 and September 2013. Inclusion criteria for this study were those patients: (i) ages were from ten to twenty years old; (ii) females; and (iii) no scoliosis operation. The average age at the time of bone density measurement was 15.2 ± 2.0 years-old (range: 10–19). Eighteen subjects were evaluated before treatment, 31 had been treated by brace. The number of subjects scored using the Risser sign as a parameter of bone growth were Grade 0: 1, Grade 1: 4, Grade 2: 1, Grade 3: 6, Grade 4: 24, and Grade 5: 13.

The average age of menarche was 12.3 ± 1.1 years-old and two subjects had not had menarche yet. Height and weight measured on the date closest to the bone density test and calculated BMI were used in the analysis. The degree of scoliosis was evaluated as the Cobb angle of major curvature from long antero-posterior standing radiographs. The mean ± SD of the Cobb angle was 39 ± 13 degrees. Using the Lenke classification, 29 cases were type1, 3 cases were type2, 1 case was type3, 0 cases was type 4, and 16 cases were type 5. This study was approved by the Institutional Review Board of Yokohama City University Hospital (B120906031) and the subjects and their parents for those underage were consented.

### Bone mineral density

All BMDs were measured using the same type DEXA machine (Hologic Corp; QRS series, Discovery A, 35 Grosby Drive Bedford, MAO1730 USA) according to the manufacturer’s instructions. Frontal views of the second to fourth lumbar vertebrae and the femoral necks of bilateral proximal femurs and bilateral proximal femurs excluding the Ward triangle were examined. BMD measurements of the proximal femur included the femoral neck, trochanter, and inter trochanter area [9].

Lumbar spine BMD measurements were compared with the normal data defined by Nishiyama and Okada [10] with differences expressed as the age- and sex- matched mean (the “Z score”) and femoral neck and total proximal femur BMD measurements were compared with normal date defined by Kalkwarf HJ et al. [11] with differences expressed as the Z score. Since lumber BMD were tended to be affected by scoliosis [12], the lowest femoral Z scores were used for analysis [9]. Since the definitions of osteopenia and osteoporosis were not clear in children, the definitions by Cassidy [13] that BMDs above −1 standard deviation (−1SD) were defined as normal, BMDs from -1SDs to -2SDs were defined as osteopenia, and the BMDs below -2SDs were defined as osteoporosis were used in this study.

### Bone metabolism markers

Serum levels of BAP as a bone formation marker and TRAP5b as a bone resorption marker were collected from 09:00 to 17:00 and stored at −80°C until assayed. The frozen samples were thawed and measurements were made immediately after thawing. According to Rauchenzauner et al. [14], the normal range at each age for BAP and TRAP5b was defined as values from −1.88SD to +1.88SD from the normal mean. For this study, subjects with values of TRAP5b above +1.88SD from the normal mean were classified as the high TRAP5b group, those with values from −1.88SD to +1.88SD were classified as the normal group, and the those with values below −1.88SD were classified as the low TRAP5b group.

## Statistics

Proportional differences were analyzed using the Chi-square test for independence. Correlations among parametric data were assessed by the Pearson’s correlation coefficient test. Student’s *T*-test was used to determine significant differences between means for two groups of data. When samples had possible unequal variances, Welch’s test was utilized. For ordinal data, the Mann–Whitney *U*-test was employed. Correlations among nonparametric data were assessed by Spearman’s correlation coefficient rank test. Statistical significance was accepted at p < 0.05.

## Results

The average BMI was 18.8 ± 2.4 kg/m^2^. Mean BMD and Z scores of each bone area are listed in Table 1. There were 17 subjects (35%) in normal (Z scores > −1), 25 subjects (51%) in osteopenia (−2 < Z score ≦ − 1), and 7 subjects (14%) in osteoporosis (Z score ≦ − 2) by using the lowest femoral Z scores. There were significant correlations between lumbar and right femoral neck BMDs (r = 0.68, p < 0.01), lumbar and right proximal femur BMDs (r = 0.79, p < 0.01), lumbar and left femoral neck BMDs (r = 0.62, p < 0.01), and lumbar and right proximal femur BMDs (r = 0.72, p < 0.01).

**Table 1:** **BMD and Z scores in AIS patients**

Lumbar spine BMD (g/cm2)	0.9 +/−0.1
Lumbar spine Z scores (±SD)	−1.2 +/−1.1
Right femoral neck BMD (g/cm2)	0.8 +/−0.1
Right femoral neck Z score (±SD)	−0.8 +/−0.9
Right proximal femur BMD (g/cm2)	0.8 +/−0.1
Right proximal femur Z score (±SD)	−0.8 +/−0.9
Left femoral neck BMD (g/cm2)	0.7 +/−0.1
Left femoral neck Z score (±SD)	−0.8 +/−0.9
Left proximal femur BMD (g/cm2)	0.8 +/−0.1
Left proximal femur Z score (±SD)	−0.9 +/−0.8
**The lowest femur Z score (±SD)**	**−1.2 +/−0.9**

BMD = bone mineral density.

Z score = difference from normal mean expressed as standard deviation.

AIS = adolescent idiopathic scoliosis.

**Figure 1:**
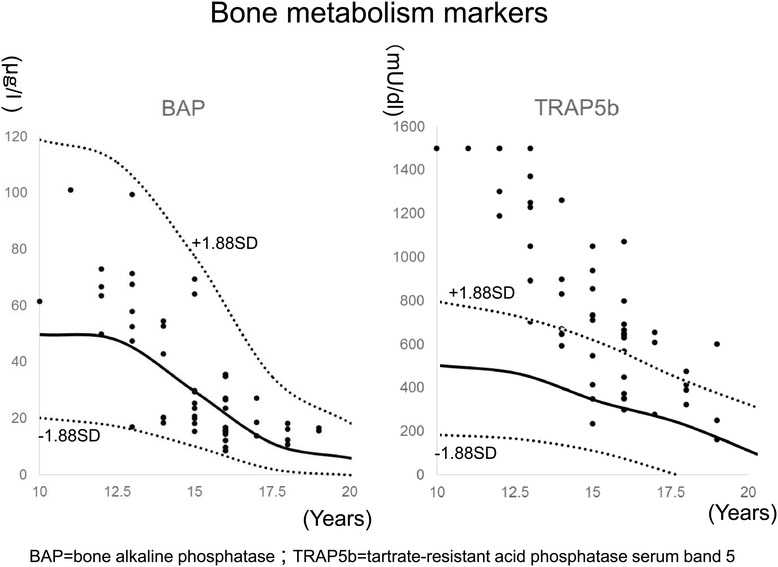
**The distribution of bone metabolism markers in female by age.** Bone alkaline phosphatase (BAP) is a marker of bone formation and tartrate-resistant acid phosphatase serum band 5 (TRAP5b) is a marker of bone resorption. Solid lines represent the mean and the dotted lines represent ± 1.88SD. Normal values were referenced according to data of Rauchenzauner et al. [14].

BAP and TRAP5b serum measurements by age are shown in Figure 1. Values of BAP were within normal limits for 100% of the subjects, while 29 subjects (59%) showed high values of TRAP5b (above +1.88 SDs) (Table 2).

**Table 2:** **Number and percentage of high, normal, and low values of BAP and TRAP5b**

***BAP (bone alkaline phosphatase)***	***N (49)***	***%***
High (above +1.88SD)	0	0
Normal (−1.88SD to +1.88SD)	49	100
Low (below −1.88SD)	0	0
***TRAP5b*** *(tartrate-resistant acid phosphatase serum band 5)*	***N (49)***	***%***
High (above +1.88SD)	29	59
Normal (−1.88SD to +1.88SD)	20	41
Low (below −1.88SD)	0	0

BAP = bone alkaline phosphatase.

TRAP5b = tartrate-resistant acid phosphatase serum band 5.

BMDs, Z scores, age of menarche, BMI, and Cobb angle were compared between the subjects with high serum values of TRAP5b and those with normal values. In the high TRAP5b group (n = 29), the age was significantly lower (p = 0.01) and BMI significantly lower (p < 0.01) than those of the normal TRAP5b group (n = 20) (Table 3). The lowest femoral Z scores of the high TRAP5b group were significantly lower (p = 0.02) than those of the normal group (Table 3).

**Table 3:** **Differences between normal and high TRAP5b groups**

	**Normal TRAP5b**	**High TRAP5b**	**p value**
	**group (N = 20)**	**group (N = 29)**	
***Age***	***16.2 +/−1.7***	***14.5 +/−2.0***	***0.01***
Menarche (age)	12.2+/−1.3	12.3 +/−1.0	0.58
***BMI (kg/cm2)***	***20.2 +/−2.2***	***17.9 +/−2.0***	***<0.01***
Cobb angle (degrees)	35.1 +/−12.2	41.1 +/−14.0	0.29
Lumbar spine Z score	−1.0 +/−1.0	−1.4 +/−1.1	0.12
***The lowest femoral Z scores***	***−0.8 +/−0.7***	***−1.4 +/−0.9***	***0.02***

TRAP5b = tartrate-resistant acid phosphatase serum band 5.

Correlations between the lowest femoral Z scores and age of menarche, BMI, bone metabolism markers (BAP, TRAP5b), and Cobb angle are shown in Table 4. The lowest femoral Z scores were positively correlated with BMI and not correlated with age of menarche, serum values of TRAP5b and BAP, and Cobb angle. The Cobb angle was positively correlated with serum values of TRAP5b, but not significantly correlated with age of menarche, BMI, values of BAP, and the lowest femoral Z score (Table 5).

**Table 4:** **Correlations between the lowest femoral Z scores and contributing factors**

	**R**	**p value**
Age of menarche	−0.25	0.10
BMI	0.34	0.02
TRAP5b	−0.26	0.07
BAP	−0.19	0.20
Cobb angle	−0.21	0.15

BMI = body mass index.

TRAP5b = tartrate-resistant acid phosphatase serum band 5.

BAP = bone alkaline phosphatase.

**Table 5:** **Correlations between Cobb angle and contributing factors**

	**R**	**p**
Age of menarche	0.28	0.06
BMI	−0.25	0.09
***TRAP5b***	***0.30***	***0.04***
BAP	0.27	0.07
The lowest femoral Z score	−0.21	0.15

BMI = body mass index; TRAP5b = tartrate-resistant acid phosphatase serum band 5.

BAP = bone alkaline phosphatase; BMD = bone mineral density.

## Discussion

The BMDs are generally evaluated by the DEXA measured in lumbar spine, femoral neck, and total proximal femur and the lowest BMDs of each area were used for analysis [9]. Because the BMDs of rotated lumbar spines changed by degrees like AIS subjects [12] and the BMDs changes by age and sexual growth in adolescent period, the lowest femoral Z score (the age- and sex- matched mean) was used for analysis.

The incidence of osteopenia in AIS has been reported to be about 30% [2-6]. In our study, 65% of AIS patients were osteopenic or osteoporotic by using the lowest femoral Z score. The higher frequency in the present study may be due to the sampling error inherent in our relatively small sample size and the several definitions of osteopenia and osteoporosis.

Previous researchers reported that low BMD in AIS was associated with delayed menarche [6] and low BMI [13]. In our study, the correlation between the lowest femoral Z scores and age of menarche was not significantly correlated. In agreement with a previous report [15], a significant positive correlation between the lowest femoral Z scores and BMI was found in our study.

Bone metabolism in AIS has not been well described. Cheung et al. measured serum concentrations of BAP and urinary concentrations of deoxypyridinoline in 621 AIS patients [7]. They reported serum concentrations of BAP in AIS patients from age13 to 15 years-old were on average 39% higher than those of age-matched controls and that urinary concentrations of deoxypyridinoline in AIS patients older than 15 years-old were 30% lower than those of age-matched controls. They concluded that AIS patients had higher bone turnover because they had 39% higher BAP concentrations. However, their finding that AIS patients had 30% lower deoxypyridinoline concentrations than age-matched controls is not consistent with their conclusion. A limitation of their study was that they used urinary deoxypyridinoline as a bone resorption marker. Urinary deoxypyridinoline is one of two pyridinum cross-links providing structural stiffness to collagen type 1 in bones and has been reported to be more affected by renal function, fasting, and hourly variations than TRAP5b [16].

Serum TRAP5b is a biomarker for osteoclastic bone resorption activity and has been reported to demonstrate little daily variation; a low variability of 14% was observed from 09:00–17:00 while urine resorption markers vary as much as 137% throughout the day. TRAP5b also demonstrated minimal response to fasting, a decrease of only 2%, whereas other serum and urine resorption markers decrease 18% during fasting [8]. Although 29 of our AIS subjects (59%) had high values for TRAP5b, no subject had a high value for BAP. These data suggest there may be high osteoclast activity but normal osteoblast activity and an imbalance in bone metabolism. Examining this imbalance in bone metabolism, Chiru reported that mean RANKL and RANKL to OPG ratios in 15 patients with AIS were increased compared to those in control subjects, suggesting high osteoclast activity and an imbalance in the RANKL/OPG system [17].

In our study, the lowest femoral Z scores were significantly low with high TRAP5b group. This result means the cause of low BMD is increased osteoclast activity as indicated by the high TRAP5b levels. This is the first report describing high values of a bone resorption marker associated with low bone density in AIS patients.

Age and BMI in the high TRAP5b group were significantly lower than those in the normal TRAP5b group. As shown in Figure 1, the normal average values of TRAP5b were decreasing slowly with age, although the average values of TRAP5b in AIS were decreasing rapidly from abnormal high values. This means abnormal bone metabolism in AIS would start before notice of scoliosis. According to low BMI in the high TRAP5b group, the BMI and BMD had been reported to be positive correlation and high TRAP5b resulted in low BMD.

Reasons for high values of TRAP5b in AIS may include low levels of sex hormones [18], lack of calcium intake from elevated levels of parathyroid hormone [7], deficiency of melatonin affecting the melatonin receptor of osteoblasts, stimulation of stem cell proliferation and cell differentiation [19,20], and deficiency of leptin, which acts on marrow stromal cells to enhance differentiation to osteoblasts [21] while inhibiting osteoclast generation [19,22]. Our study did not assess melatonin, leptin, sex hormones, calcium intake, and other causes of high values of TRAP5b. These factors may be intricately intertwined.

A significant relationship between severity of scoliosis and BMD has been reported [5], while another study found no relationship [1]. In a histomorphometric study, pinealectomy in broiler chicken model was reported to induce high turnover osteoporosis, which might contribute to the development of scoliosis in the chicken [23].

Lu et al. reported that anti- osteoporosis treatment could improve bone strength, prevent osteoporosis and rebalance the OPG- RANK-RANKL system, which might help to prevent curve progression in AIS [24]. In our study, the Cobb angle was positively correlated with TRAP5b, but the correlation coefficient was not as strong as the value of 0.3 and the causes of scoliosis aggravation would be multi factors.

Our study has limitations, including small sample size, varying age of subjects. In addition, data from normal controls reported from previous studies were used because our study did not have normal controls.

## Conclusion

This study assessed bone mineral density and bone metabolism in AIS patients using the bone metabolism markers, BAP and TRAP5b. Sixty-five percent of AIS patients had osteopenia or osteoporosis and 59% of AIS patients had high values for TRAP5b. The AIS patients with high values of TRAP5b had lower Z scores than those with normal values of TRAP5b. Higher rates of bone resorption are associated with low bone density in AIS patients.
